# Supplementary material for: Dietary Patterns in Relation to Prospective Sleep Duration and Timing among Mexico City Adolescents
Source: Nutrients. 2020 Jul 31;12(8):2305. doi: 10.3390/nu12082305 (PMC7468850; doi:10.3390/nu12082305)
Supplement: Supplementary file 1 [file nutrients-12-02305-s001.zip › Supplemental figure 1.docx]

**Supplemental Figure 1.** Meat & Starchy pattern scores in relation to social jetlag (difference between weekend and weekday midpoint) at follow-up

N=488

R=0.12; P value=0.01
