# Supplementary material for: Dietary Patterns in Relation to Prospective Sleep Duration and Timing among Mexico City Adolescents
Source: Nutrients. 2020 Jul 31;12(8):2305. doi: 10.3390/nu12082305 (PMC7468850; doi:10.3390/nu12082305)
Supplement: Supplementary file 1 [file nutrients-12-02305-s001.zip › Supplemental Table 1.docx]

| **Supplemental Table 1**. Average food intakes (servings/day) according to categories (quartile 1 versus quartile 4) of dietary patterns | | | | | | | | | | | | |
| --- | --- | --- | --- | --- | --- | --- | --- | --- | --- | --- | --- | --- |
|  | Plant-based & lean proteins | | | | Meat & starchy | | | | Eggs, milk & refined grain | | | |
| Food group | Q1,  Mean (SD) | | Q4,  Mean (SD) | | Q1,  Mean (SD) | | Q4,  Mean (SD) | | Q1,  Mean (SD) | | Q4,  Mean (SD) | |
|  |  | |  | |  | |  | |  | |  | |
| other vegetables | 0.18^1^ | (0.31) | 1.10 | (0.73) | 0.54 | (0.52) | 0.56 | (0.54) | 0.53 | (0.65) | 0.55 | (0.54) |
| cruciferous vegetables | 0.01 | (0.04) | 0.30 | (0.46) | 0.12 | (0.19) | 0.08 | (0.17) | 0.14 | (0.37) | 0.10 | (0.25) |
| fruit | 0.73 | (0.55) | 2.33 | (1.80) | 1.45 | (1.14) | 1.61 | (1.47) | 1.14 | (1.16) | 1.73 | (1.54) |
| dark yellow vegetables | 0.05 | (0.09) | 0.39 | (0.33) | 0.22 | (0.26) | 0.20 | (0.21) | 0.19 | (0.25) | 0.23 | (0.24) |
| leafy vegetables | 0.15 | (0.17) | 0.70 | (0.45) | 0.38 | (0.39) | 0.36 | (0.35) | 0.38 | (0.44) | 0.34 | (0.36) |
| tomatoes | 0.05 | (0.14) | 0.40 | (0.35) | 0.23 | (0.29) | 0.15 | (0.25) | 0.20 | (0.29) | 0.18 | (0.25) |
| chicken | 0.24 | (0.26) | 0.55 | (0.37) | 0.32 | (0.24) | 0.49 | (0.35) | 0.43 | (0.40) | 0.36 | (0.29) |
| fish | 0.08 | (0.13) | 0.28 | (0.30) | 0.18 | (0.26) | 0.18 | (0.23) | 0.15 | (0.23) | 0.16 | (0.19) |
| avocado | 0.13 | (0.19) | 0.39 | (0.45) | 0.25 | (0.34) | 0.31 | (0.42) | 0.23 | (0.33) | 0.21 | (0.29) |
| soup | 0.13 | (0.21) | 0.35 | (0.36) | 0.17 | (0.22) | 0.37 | (0.41) | 0.30 | (0.36) | 0.23 | (0.28) |
| high-fat dairy | 0.42 | (0.38) | 0.87 | (0.52) | 0.45 | (0.30) | 0.87 | (0.59) | 0.55 | (0.40) | 0.69 | (0.55) |
| water & unsweetened drinks | 1.67 | (1.68) | 3.27 | (1.94) | 3.06 | (1.89) | 2.05 | (1.84) | 1.96 | (1.93) | 2.76 | (1.92) |
| chips | 0.36 | (0.37) | 0.22 | (0.28) | 0.09 | (0.15) | 0.48 | (0.36) | 0.31 | (0.33) | 0.19 | (0.25) |
| refined grains | 1.57 | (0.99) | 1.73 | (0.96) | 1.11 | (0.63) | 2.27 | (1.03) | 1.21 | (0.78) | 2.13 | (1.09) |
| sugar-sweetened beverages | 2.37 | (1.61) | 1.80 | (1.33) | 1.26 | (1.05) | 2.75 | (1.67) | 2.66 | (1.77) | 1.71 | (1.32) |
| processed meat | 0.29 | (0.42) | 0.18 | (0.24) | 0.11 | (0.15) | 0.41 | (0.43) | 0.20 | (0.23) | 0.26 | (0.38) |
| Mexican foods | 0.25 | (0.27) | 0.32 | (0.30) | 0.14 | (0.17) | 0.45 | (0.39) | 0.28 | (0.30) | 0.29 | (0.28) |
| potato & fried plantains | 0.13 | (0.20) | 0.22 | (0.23) | 0.09 | (0.12) | 0.28 | (0.27) | 0.22 | (0.26) | 0.15 | (0.17) |
| sweets/dessert | 0.74 | (0.58) | 1.16 | (0.89) | 0.69 | (0.60) | 1.14 | (0.79) | 0.88 | (0.63) | 0.88 | (0.65) |
| pork | 0.15 | (0.22) | 0.14 | (0.21) | 0.06 | (0.10) | 0.23 | (0.29) | 0.21 | (0.30) | 0.07 | (0.11) |
| legumes | 0.48 | (0.54) | 0.56 | (0.45) | 0.26 | (0.33) | 0.70 | (0.56) | 0.35 | (0.42) | 0.57 | (0.51) |
| corn tortillas | 0.78 | (0.35) | 0.78 | (0.40) | 0.67 | (0.38) | 0.88 | (0.33) | 0.65 | (0.39) | 0.85 | (0.29) |
| milk | 1.43 | (1.07) | 1.47 | (0.98) | 1.60 | (1.03) | 1.22 | (0.94) | 0.48 | (0.44) | 2.33 | (0.69) |
| sweetened milk | 1.02 | (1.47) | 0.46 | (0.71) | 0.62 | (0.95) | 0.70 | (1.08) | 0.22 | (0.36) | 1.50 | (1.58) |
| mayonnaise or margarine | 0.18 | (0.25) | 0.20 | (0.26) | 0.15 | (0.21) | 0.29 | (0.35) | 0.10 | (0.17) | 0.36 | (0.37) |
| egg | 0.24 | (0.23) | 0.30 | (0.24) | 0.23 | (0.24) | 0.32 | (0.24) | 0.18 | (0.20) | 0.38 | (0.25) |
| fast food | 0.38 | (0.35) | 0.51 | (0.40) | 0.36 | (0.29) | 0.52 | (0.38) | 0.37 | (0.34) | 0.54 | (0.41) |
| natural juice | 0.07 | (0.17) | 0.17 | (0.28) | 0.09 | (0.19) | 0.16 | (0.28) | 0.06 | (0.15) | 0.23 | (0.28) |
| corn on the cob | 0.05 | (0.12) | 0.12 | (0.20) | 0.04 | (0.09) | 0.13 | (0.24) | 0.13 | (0.24) | 0.07 | (0.14) |
| whole grains | 0.01 | (0.06) | 0.13 | (0.24) | 0.08 | (0.19) | 0.08 | (0.20) | 0.05 | (0.14) | 0.08 | (0.21) |
| yogurt | 0.32 | (0.44) | 0.54 | (0.64) | 0.34 | (0.38) | 0.45 | (0.53) | 0.37 | (0.48) | 0.44 | (0.49) |
| beef | 0.13 | (0.18) | 0.23 | (0.26) | 0.16 | (0.16) | 0.23 | (0.28) | 0.21 | (0.26) | 0.17 | (0.18) |
| butter | 0.06 | (0.12) | 0.08 | (0.23) | 0.03 | (0.09) | 0.11 | (0.26) | 0.07 | (0.15) | 0.06 | (0.16) |
|  |  |  |  |  |  |  |  |  |  |  |  |  |

^1^ Values are energy-adjusted average servings/day
